# 成功使用白介素-6抗体托珠单抗控制肺腺癌顽固性肿瘤热1例病例报道

**DOI:** 10.3779/j.issn.1009-3419.2026.102.01

**Published:** 2026-01-20

**Authors:** Ying LI, Chunhui LI, Bin LI

**Affiliations:** ^1^410008 长沙，中南大学湘雅医院内科（李颖）; ^1^Department of Internal Medicine; ^2^感染科（李春辉）; ^2^Department of Infectious Diseases; ^3^肿瘤内科（李斌）; ^3^Department of Oncology, Xiangya Hospital, Central South University, Changsha 410008, China

**Keywords:** 肺肿瘤, 肿瘤热, 白介素-6, 托珠单抗, Lung neoplasms, Paraneoplastic fever, Interleukin-6, Tocilizumab

## Abstract

发热是肺癌较为常见的症状，但以合并感染多见。本文在国内首次报道了1例肺腺癌患者在使用一线治疗疾病进展再次活检后出现不明原因持续1个月以上的发热；经全面抽血、病原学检测及影像学检查均不支持感染性发热、结缔组织疾病发热、药物热等，继而诊断为肿瘤热；使用布洛芬后，患者发热仍难以控制，静脉给药1次托珠单抗12 h后即成功控制患者发热，并维持体温正常至随访日共45天（2025年8月21日至10月4日）。本例报道为探讨肿瘤热发病机制及治疗策略提供了初步的实践经验。

## Abstract (en)

Fever is a common symptom of lung cancer, but most are induced by infection. This is the first report of a patient with lung adenocarcinoma who developed a fever of unknown origin for more than 1 month after repeat biopsy on the progression of first-line treatment in China. Comprehensive blood tests, the well-known pathogens were fully examined, and imaging studies failed to support infectious fever, connective tissue disease-related fever, drug fever, or other etiologies, leading to the diagnosis of tumor fever. While the fever remained refractory to Ibuprofen, it was successfully controlled 12 h after a single intravenous administration of Tocilizumab, with the patient maintaining a normal body temperature for 45 days, from August 21 to October 4, 2025. This case report primarily provides the practice for exploring the tumor fever.

原发性支气管肺癌是发病率和死亡率均最常见的恶性肿瘤之一^[1]^，除咳嗽、咯血、呼吸困难、胸痛等症状^[2]^外；部分患者可出现发热^[3]^，常为感染所致。目前临床上关于肿瘤热的治疗以抗肿瘤治疗为主，辅以萘普生或其他非甾体抗炎药退热^[4]^。然而当患者已使用抗肿瘤治疗及非甾体抗炎药退热后仍反复高热是临床上治疗的难点。我们报道1例表皮生长因子受体（epidermal growth factor receptor, EGFR）突变阳性晚期肺腺癌患者，初始予EGFR-酪氨酸激酶抑制剂（EGFR-tyrosine kinase inhibitors, EGFR-TKIs）靶向治疗；后病情进展行纵隔淋巴结穿刺，并于穿刺后1周出现发热、肿块增大及肿瘤相关性心包、胸腔积液。更改抗肿瘤方案后，影像学示肿块稳定、积液吸收，但患者反复高热（最高39.8 ^o^C）持续1个月，布洛芬退热效果短暂。经全面检查排除感染、结缔组织病、药物等相关发热后，诊断为肿瘤热。予托珠单抗静脉注射后退热，至随访日体温正常45天（2025年8月21日至10月4日）。本例报告为探讨肿瘤热发病机制及治疗策略提供了初步的实践经验。

## 1 病例资料

患者女，49岁，2024年7月27日因“间断头晕头痛8月余，左侧肢体乏力摔倒2天”于中南大学湘雅医院首次就诊，完善胸部计算机断层扫描（computed tomography, CT）、头部磁共振成像示右肺下叶背段类圆形肿块影（大小约46 mm×46 mm），纵隔及右肺门内肿大淋巴结（最大短径约14 mm），双侧胸腔积液；右额叶可见团块状等-长T1等-长T2信号灶；临床诊断：肺癌脑转移；予以显微镜下行幕上深部病变切除术及术后瘤床的伽马刀放疗；结合术后病理、基因检测回报，诊断为：肺腺癌，cT2bN2M1b IVA期[EGFR外显子19缺失突变，程序性死亡配体1（programmed cell death ligand 1, PD-L1）<1%]，脑转移癌（右额叶）。于2024年8月30日开始行伏美替尼治疗，1个月后疗效评价为部分缓解。

2025年3月开始，患者CT示纵隔淋巴结逐渐增大。5月21日患者CT示右肺肿块及纵隔淋巴结均增大（33 mm），考虑肿瘤进展，于6月30日完善经支气管镜超声引导下纵隔淋巴结（第4组）穿刺，病理示低分化腺癌。穿刺后1周（7月7日）出现发热（最高39.7 ^o^C），多于下午及晚上出现，伴咳嗽咳痰，当地医院血液检测：白细胞（white blood cell, WBC）6.65×10^9^/L、中性粒细胞（neutrophil, N）5.25×10^9^/L、血沉（erythrocyte sedimentation rate, ESR）120 mm/h、超敏C反应蛋白（hypersensitive C-reactive protein, hs-CRP）185 mg/L、降钙素原（procalcitonin, PCT）0.07 ng/mL、支原体IgG（+）、IgM（-）、血培养阴性；不排除感染，予以阿奇霉素抗感染及布洛芬退热，但患者发热症状反复。7月12日检查示：WBC 13.1×10^9^/L、N 11.97×10^9^/L、ESR 120 mm/h、CRP 154 mg/L、PCT 0.93 ng/mL、白介素-6（interleukin-6, IL-6）68.2 pg/mL、IL-1β 18.9 pg/mL、肿瘤坏死因子-α（tumor necrosis factor-α, TNF-α）20.3 pg/mL升高，大便酵母菌100%（考虑使用抗生素后的菌群失调），痰培养示肺炎克雷伯弱阳性、偶见白色念珠菌，输血前四项、G+GM试验、结核感染T细胞、肺炎支原体、巨细胞病毒+EB病毒、新冠病毒、甲/乙流感病毒、呼吸道腺病毒、人类鼻病毒、呼吸道合胞病毒、痰液抗酸染色阴性。7月14日患者上述症状加重并出现晕厥，考虑I型呼吸衰竭。紧急入重症监护病房治疗，经验性予哌拉西林钠他唑巴坦（7月15日至18日）抗感染，7月15日复查肺部CT示：右肺病灶进一步增大（38 mm×25 mm），纵隔淋巴结增大明显（70 mm）并浸润心包及胸膜，出现大量心包积液及双侧胸水（图1A）。于7月18日予二线抗肿瘤治疗，静脉予贝伐珠单抗0.3 g、培美曲塞0.5 g及卡铂0.4 g方案，辅以抗生素抗感染。患者胸痛及气促明显好转，体温、WBC、N恢复至正常。7月30日患者再次出现发热，无明显规律，体温逐日升高（最高39.4 ^o^C），发热前无寒战、无咳嗽咳痰、无皮疹关节疼痛，布洛芬退热效果短暂，检查示：WBC、N正常。8月10日再次入我院治疗，予原方案抗肿瘤后，患者仅退热1天后再发高热且体温较前增高（最高39.8 ^o^C），多为晨起、午后出现，伴有畏寒，无寒战（图2），美国东部肿瘤协作组体能状态（Eastern Cooperative Oncology Group performance status, ECOG PS）评分3分，检查示：WBC、N正常，ESR 103 mm/h、hs-CRP 289.38 mg/mL、PCT 0.21 ng/mL、IL-6 159 pg/mL（图3）、IL-1β 41 pg/mL、TNF-α 17 pg/mL升高，抗核抗体阳性（1:80），ANA谱示Ro52阳性，骨髓细胞学示骨髓增生活跃，粒系活跃、分叶核增加、未见中毒颗粒；肺炎支原体IgM抗体、新冠病毒、细小病毒B19 IgM抗体、血培养、痰培养、尿培养、骨髓培养、血管炎三项、血清蛋白及免疫固定电泳、抗中性粒细胞抗体、抗环瓜氨酸肽抗体阴性。胸+腹+髂骨增强CT示：纵隔肿块稳定，双侧胸水和心包积液已吸收，未见骨骼异常（图1B）。多次感染控制中心会诊，予经验性抗感染，先后使用哌拉西林他唑巴坦钠、多西环素无效；发热程度加重，频次增加。8月19日复查示：ESR 100 mm/h、hs-CRP 253.08 mg/mL、PCT 0.21 ng/mL、IL-1β 5.56 pg/mL、TNF-α 7.14 pg/mL、IL-6 192 pg/mL（图3）。多学科会诊讨论：考虑癌症患者发热>37.8 ^o^C，持续2周以上，PCT正常、IL-6进行性增加，无感染证据，不支持感染性发热，无结缔组织疾病、过敏反应、药物热可能，抗生素使用1周以上无效，布洛芬退热短暂有效，诊断为肿瘤热。考虑IL-6进行性升高，使用IL-6受体（IL-6 receptor, IL-6R）单克隆抗体托珠单抗治疗。肿瘤热属于超适应证用药，经和患者家属进行充分沟通，并签署药品未注册同意书、高风险谈话、患者知情同意书和伦理审批表（伦理号：2026020277）后，使用托珠单抗；在静脉给药托珠单抗12 h后，患者的体温即恢复正常（图2），随访至10月4日（45天），均未出现发热；同时患者精神状态逐渐好转，体重增加，ECOG PS评分1分；复查肺部增强CT示肿瘤病灶缩小，淋巴结大小稳定；IL-6呈药效学升高（图3）。

**图1:**
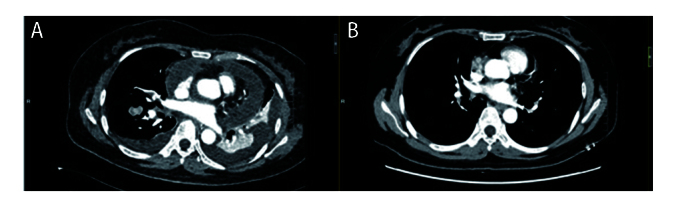
患者胸部增强CT。A：右肺下叶背段肿块灶较前稍增大，肺窗测量较大层面大小约38 mm×25 mm。双肺可见散在条片状、斑片状密度增高影，双侧胸腔积液、心包积液；B：右肺下叶背段肿块较前稍缩小，肺窗测量较大层面大小约35 mm×23 mm，内空洞较前增大。双侧胸腔积液、心包积液较前已吸收。无胸水、心包积液、癌性淋巴管炎。

**图2:**
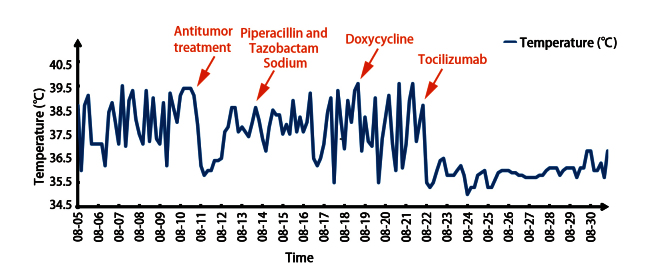
患者8月体温表。8月10日予以抗肿瘤治疗：贝伐珠单抗0.3 g+培美曲塞0.5 g+卡铂0.4 g；8月13日至18日予以哌拉西林他唑巴坦钠4.5 g q8h抗感染；8月18日至21日予以多西环素0.1 g bid抗感染；8月21日予以托珠单抗464 mg（8 mg/kg）。

**图3:**
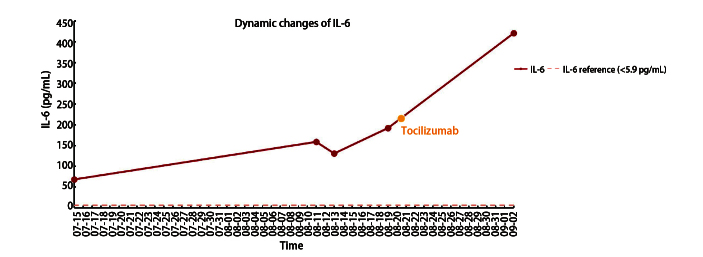
白介素-6的动态变化。图中红色点为对应日期时测量的IL-6值，红色连线为5次IL-6测量值之间的连线，橙色点处为托珠单抗注射时间8月21日。

## 2 讨论

目前，肿瘤热的发生机制不明^[4]^，可能与恶性肿瘤释放炎症因子有关，如IL-1、IL-6及TNF-α等可直接作用于下丘脑体温调节中枢，导致体温调定点上移，从而引起发热^[5]^。研究^[6]^表明，肿瘤细胞可通过多种途径释放炎症因子，如旁分泌IL-1β（成纤维细胞、树突状细胞和单核细胞等）、IL-10（M2型巨噬细胞、调节性T细胞等），或自分泌与旁分泌同时释放IL-6。本例患者于6月行支气管镜活检术后，7月即出现发热，伴呼吸困难、胸痛，考虑与肿瘤快速进展或穿刺创伤诱导炎症反应有关，行抗肿瘤治疗后发热好转。8月患者再次发热，炎症因子升高明显（尤其是IL-6），排除了感染性发热、结缔组织疾病、过敏反应、药物热可能，同时患者使用布洛芬可有效退热，但效果短暂，与既往使用萘普生实验诊断肿瘤热效果类似，两者均为非甾体类抗炎药，因此诊断为肿瘤热，IL-6R单抗托珠单抗可有效退热。

IL-6导致机体发热的作用机制比较明确^[5,6]^；研究^[7]^发现，在癌症细胞快速进展时，释放大量IL-6导致血液浓度升高和全身炎症症状。IL-6诱导环氧合酶2（cyclooxygenase 2, COX-2）的合成，促进免疫细胞、血管内皮细胞、神经细胞中的花生四烯酸转变成前列腺素E2（prostaglandin E2, PGE2），PGE2可通过血脑屏障进入中枢，或直接由中枢产生作用于下丘脑的体温调节中枢，引起体温调定点上移，导致发热^[5]^；研究^[8]^发现，患者在肿瘤被切除后，体温和IL-6水平均恢复正常。本例患者动态监测多项血清炎症因子的表达水平，结果发现，仅IL-6进行性增高，其他包括IL-1β、IL-10、TNF-α均下降至正常水平左右，这些结果提示发热可能与IL-6的作用有关。

托珠单抗作为第一个已上市的人源化抗IL-6R的单克隆抗体，已广泛用于多种自身免疫性疾病^[10]^。托珠单抗不仅与细胞膜上IL-6R结合，减少经典的信号传导通路，也可与大量的游离IL-6R（soluble IL-6R, sIL-6R）特异性结合，减少了大量sIL-6R对全身多处糖蛋白130（glycoprotein, gp130）的反式信号传导，完全阻断IL-6/IL-6R/gp130复合物的形成及其激活的下游信号级联，抑制促炎细胞因子和趋化因子基因表达，减少了炎症细胞的活化和炎症介质的释放，从根源上遏制了炎症反应的级联放大^[5]^。近期Blya等^[9]^研究发现，托珠单抗可有效控制实体瘤（肉瘤、肺癌和乳腺癌）的肿瘤热，有效率为100%。托珠单抗未来可能作为控制肿瘤热的有效手段。

总之，迄今为此，我们在国内首次报道了托珠单抗控制肺腺癌患者顽固性肿瘤热；综合文献和本病例发现，IL-6在介导肿瘤热、恶病质及肿瘤细胞药物耐药等方面起着重要作用；而拮抗IL-6的治疗有望成为增敏抗肿瘤疗效、控制症状、改善生活质量的重要治疗措施，值得扩大样本进行前瞻性临床研究。
